# CKD Associates with Cognitive Decline in Middle-Aged and Older Adults with Long-Standing Type 1 Diabetes

**DOI:** 10.34067/KID.0000000000000178

**Published:** 2023-06-09

**Authors:** Minesh Khatri, Christopher M. Ryan, Xiaoyu Gao, Ian H. de Boer, Barbara H. Braffett, Mark Molitch, Amy B. Karger, Gayle M. Lorenzi, Pearl Lee, Victoria R. Trapani, John M. Lachin, Alan M. Jacobson

**Affiliations:** 1 NYU Long Island School of Medicine, Mineola, New York; 2 University of Pittsburgh, Pittsburgh, Pennsylvania; 3 Biostatistics Center, The George Washington University, Rockville, Maryland; 4 Division of Nephrology, University of Washington, Seattle, Washington; 5 Division of Endocrinology, Metabolism and Molecular Medicine, Department of Medicine, Northwestern University Feinberg School of Medicine, Chicago, Illinois; 6 University of Minnesota Twin Cities, Twin Cities, Minnesota; 7 University of California San Diego, La Jolla, California; 8 Department of Internal Medicine, University of Michigan, Ann Arbor, Michigan

**Keywords:** cognition, type 1 diabetes, CKD

## Abstract

**Key Points:**

- We found that development of both albuminuria and reduced eGFR was associated with clinically significant cognitive decline, particularly in the psychomotor and mental efficiency domain.
- There was also a significant interaction between worsened albuminuria and eGFR, the combination of which augmented cognitive deficits.
- A more comprehensive longitudinal phenotype of albuminuria showed that regressed albuminuria did not associate with worsened cognitive decline, as opposed to persistent albuminuria.

**Background:**

Individuals with CKD or type 1 diabetes (T1D) are at risk for cognitive decline, but it is unclear whether these associations are with albuminuria, eGFR, or both.

**Methods:**

We examined the longitudinal relationships between CKD and change in cognition in 1051 participants with T1D in the Diabetes Control and Complications Trial and its follow-up, the Epidemiology of Diabetes Interventions and Complications study. Albumin excretion rate and eGFR were measured every 1–2 years. Three cognitive domains were assessed repeatedly over a 32-year period: immediate memory, delayed memory, and psychomotor and mental efficiency. Associations between cognitive function and CKD were assessed: (*1*) longitudinally and (*2*) in models using eGFR and albuminuria measurements over the first 15–20 years with subsequent change in cognitive function over the ensuing 14 years (when decline in cognition was greatest).

**Results:**

In fully adjusted longitudinal analyses, the magnitude of decline in the psychomotor and mental efficiency domain score was associated with eGFR <60 ml/min per 1.73 m^2^ (*β* −0.449; 95% confidence interval [CI], −0.640 to −0.259) and sustained albumin excretion rate 30 to <300 mg/24 hours (*β* −0.148; 95% CI, −0.270 to −0.026). This was equivalent to a decrease associated with approximately 11 and 4 years of aging, respectively. In analyses focused on changes in cognition between study years 18 and 32, eGFR <60 ml/min per 1.73 m^2^ was associated with reduced psychomotor and mental efficiency (*β* −0.915; 95% CI, −1.613 to −0.217).

**Conclusions:**

In T1D, development of CKD was associated with a subsequent reduction on cognitive tasks requiring psychomotor and mental efficiency. These data highlight the need for increased recognition of risk factors for neurologic sequelae in patients with T1D, as well as preventive and treatment strategies to ameliorate cognitive decline.

## Graphical abstract

**Figure:**
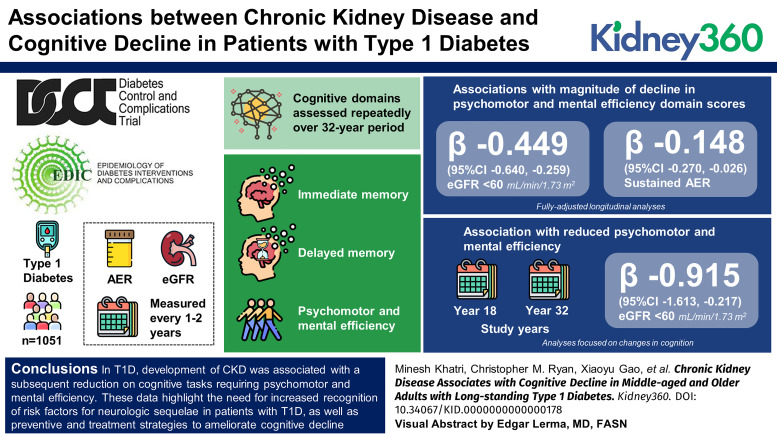

## Introduction

CKD has significant neurological and neurocognitive sequelae. In the general population, CKD is a potent independent risk factor for stroke,^1^ is associated with cerebral atrophy^2^ and white matter disease,^3^ and is correlated with poorer performance on measures of learning, memory, executive functioning, and rapid responding.^4^ While the most profound clinical findings are seen with ESKD, where up to one-third of patients on dialysis may have dementia,^5^ even mild-to-moderate CKD has been linked to cognitive impairment.^6^ Most, but not all,^7^ studies have found correlations between cognition function and the severity of reduced eGFR and/or the degree of albuminuria.^5^

Cognitive impairment has also been reported in individuals with type 1 diabetes (T1D), independent of kidney function.^8^ The strongest predictors of cognitive decline have included higher glycated hemoglobin levels, episodes of severe hypoglycemia, elevated systolic BP, and the presence of microvascular complications.^9^ A substantial proportion of individuals with T1D also have, or will develop, CKD,^10,11^ but little is known about which parameters of kidney disease are independently associated with an increased risk of neurocognitive dysfunction in T1D over time, particularly because these individuals reach older adulthood and experience other comorbid metabolic and biomedical conditions that may also affect brain structure and function.

The Diabetes Control and Complications Trial (DCCT) and its follow-up, the Epidemiology of Diabetes Interventions and Complications (EDIC) study, provide an excellent opportunity to examine relationships between CKD and cognition over time in a very large well-phenotyped cohort of middle-aged to older adults with T1D who have had detailed biomedical and neurocognitive assessments performed repeatedly for more than 30 years. Previous results from the DCCT/EDIC study have demonstrated modest associations between measures of cognition—particularly those assessing psychomotor and mental efficiency—and advanced kidney complications (broadly defined as a serum creatinine >2.0 mg/dl or requiring dialysis or kidney transplant), which persisted after adjustment for multiple key biomedical and demographic variables.^12^ However, at 18 years of follow-up, only 10% of the cohort met the criteria for advanced kidney complications, and analyses of the relationship between less severe kidney disease and cognition were not performed. When the entire cohort was reassessed approximately 14 years later, not only had a five-fold decline occurred in measures of psychomotor and mental efficiency (Z-score declined from −0.24 to −1.36), but a larger proportion of participants had evidence of kidney disease.

The goals of this analysis were to (*1*) evaluate the association between the severity and persistence (or transience) of albuminuria and the development of cognitive dysfunction in middle-aged and older adults with long duration T1D, (*2*) examine whether mild-to-moderately reduced eGFR affects cognitive function over time, (*3*) determine whether there is an interaction between albuminuria and eGFR for cognitive change, and (*4*) identify the temporal course of these effects in individuals with T1D followed over a 32-year period.

## Methods

### Study Population

The DCCT was a randomized controlled clinical trial which enrolled 1441 individuals with T1D between 1983 and 1989.^13^ Methods have been described elsewhere in detail.^14^ In brief, participants aged 13–39 years were randomly assigned to receive intensive or conventional diabetes therapy and followed for the development of diabetes-related complications. The intensive treatment regimen aimed to achieve glucose control and glycated hemoglobin levels as close to the nondiabetic range as safely possible. Two cohorts were included in DCCT: (*1*) a primary prevention cohort of individuals with diabetes duration 1–5 years, albumin excretion rate (AER) <40 mg/24 hours, and no retinopathy and (*2*) a secondary intervention cohort of individuals with diabetes duration 1–15 years, AER <200 mg/24 hours, and mild-to-moderate diabetic retinopathy. Exclusion criteria included a history of hypertension, hyperlipidemia, severe diabetic complications or medical conditions, or a serum creatinine >1.2 mg/dl or creatinine clearance <100 ml/min per 1.73 m^2^. The DCCT was stopped 1 year ahead of schedule after an average of 6.5 years of follow-up and demonstrated the beneficial effects of intensive diabetes therapy in reducing the development and progression of complications, compared with conventional therapy. Subsequently, all participants were taught intensive therapy, and 96% of participants enrolled in the follow-up observational study, EDIC.^15^ Cognitive testing was performed serially as part of both DCCT and EDIC; 1051 participants who completed the most recent cognitive assessment after an average of 32 years of follow-up in 2018–2019 (83% of all survivors) were included in this analysis.^8^

### Assessment of Kidney Function and Disease

Albuminuria was assessed annually during DCCT and every other year in EDIC.^16^ From DCCT baseline through study year 24, albuminuria was calculated using 4-hour timed urine collections. Starting in study year 25, albuminuria was estimated using spot urine samples.^17^ Serum creatinine was measured annually, and the 2009 Chronic Kidney Disease Epidemiology Collaboration formula was used to calculate eGFR.^18^ Until 2007, serum creatinine was measured with the use of the Jaffe procedure. Thereafter, creatinine was measured by an enzymatic method that produced values traceable to the isotope dilution mass spectrometry values assigned by the National Institute of Standards and Technology. We calibrated creatinine results generated before 2007 to the isotope dilution mass spectrometry-traceable values obtained with the enzymatic method.^19^ In addition to modeling these parameters as quantitative exposure variables, we also categorized them into clinically relevant disease stages to capture the evolution of kidney disease over the course of time. Each participant was categorized into one of four mutually exclusive and time-varying albuminuria groups (normoalbuminuria, sustained AER 30 to <300 mg/24 hours, regressed AER 30 to <300 mg/24 hours and AER >300 mg/24 hours) as previously described.^20^ Participants were classified as having persistent normoalbuminuria (<30 mg/24 hours) unless they developed sustained AER 30 to <300 mg/24 hours (that persisted over two consecutive time points) or AER >300 mg/24 hours (at one or more time points). Those with sustained AER 30 to <300 mg/24 hours which then normalized on two consecutive time points were considered to have regressed AER 30 to <300 mg/24 hours. If participants with regressed AER 30 to <300 mg/24 hours then subsequently went on to develop sustained AER 30 to <300 mg/24 hours or AER >300 mg/24 hours, they were reclassified accordingly. Few participants had regressed AER >300 mg/24 hours, and thus, AER >300 mg/24 hours at any point was considered a final classification. eGFR was also modeled both quantitatively and categorically on the basis of lowest achieved value at two consecutive points during the study (≥90, 75–89, 60–74, and <60 ml/min per 1.73 m^2^).

### Cognitive Testing

Participants underwent cognitive testing at baseline and follow-up years 2, 5, 18, and 32. All participants had cognitive testing at baseline and at year 32, although they did not all necessarily have cognitive testing at other time points. The testing procedure has been described elsewhere.^21^ The most recent assessment in 2018–2019 was limited to the domains of psychomotor and mental efficiency, which has been shown to be particularly affected by hyperglycemia,^22^ as well as memory. Immediate memory was tested using the Logical Memory subtest of the Weschler Memory Scale^23^ and the Wechsler Adult Intelligence Scale Digit Symbol Substitution Test.^24^ Delayed recall was assessed by the ability to recall two logical memory stories after a 10–15 minute delay. Psychomotor and mental efficiency were evaluated by tests of verbal fluency, the Wechsler Adult Intelligence Digit Symbol Substitution Test, the time to complete Trail Making Part B, and the time to complete the Grooved Pegboard exercise with the dominant and nondominant hands.^25^ Additional details of cognitive testing can be found in Supplemental Table 1. Blood glucose levels were checked immediately before testing, and testing was delayed if the glucose was <70 mg/dl; testing commenced after oral carbohydrate intake once the glucose was >90 mg/dl. For each test, Z-scores were calculated for individual participants on the basis of the mean and SD of domain-specific scores for the entire cohort at DCCT baseline. Positive Z-scores indicate relative improvement, while negative scores reflect relative deterioration compared with the overall cohort at DCCT baseline.

All participants provided informed consent, and the study was approved by the institutional review boards at all participating centers.

### Other Key Biomedical Covariates

BP and hemoglobin A1c (HbA1c) were measured quarterly during DCCT and annually during EDIC. Treatment complications including hypoglycemia resulting in coma or seizure and ophthalmologic outcomes (proliferative diabetic retinopathy, clinically significant macular edema), clinical neuropathy, cardiovascular autonomic neuropathy, and any cardiovascular disease were assessed regularly and described elsewhere.^15,26–28^

Covariates used for analyses were either fixed at baseline or time-dependent, either concurrent with the cognitive function assessment or at the last visit before each assessment. Time-weighted mean covariates (such as mean HbA1c) were computed using weights determined by the time interval between measurements.

### Statistical Analyses

Longitudinal analyses examined the association between markers of kidney function and change in cognitive functioning during three discrete time intervals: (*1*) between DCCT baseline and each cognitive testing time point (follow-up years 2, 5, 18, and 32); (*2*) changes between 5 and 18 years of follow-up, and (*3*) changes between 18 and 32 years of follow-up. These intervals were chosen based on cognitive testing only being available at these time points, which provided a long-term window into cognitive change over time.

The associations between individual markers of kidney function and change in cognition were assessed using longitudinal mixed models, accounting for repeated measurements. Comprehensive fully adjusted multivariate regression models were evaluated for each cognitive domain with covariates significant at *P* < 0.05 selected through a backwards elimination modeling technique, as described previously.^8^ We used risk factors for each cognitive domain identified in those models here. All models were adjusted for time (as a class variable) and baseline kidney function. The immediate memory domain additionally adjusted for years of education. The delayed recall domain additionally adjusted for years of education and cardiovascular autonomic neuropathy. The psychomotor and mental efficiency domain additionally adjusted for years of education, mean updated HbA1c, any hypoglycemia resulting in coma, mean updated systolic BP, mean updated pulse, any proliferative diabetic retinopathy, any clinically significant macular edema, any confirmed clinical neuropathy, any cardiovascular autonomic neuropathy, and any cardiovascular disease. Associations between cognitive function and overall categorical albuminuria or eGFR groups were assessed using an F-test. Linear regression models were also used to assess the effect of kidney function and other covariates on change in cognition between study years 5 and 18, adjusting for covariates at year 5, as well as between years 18 and 32, adjusting for covariates at year 18. Model-based estimates (Z-scores) for the effect of eGFR on cognitive function at varying levels of albuminuria were obtained from fully adjusted longitudinal mixed models with an interaction term between albuminuria and eGFR, by fixing covariates (except for albuminuria and eGFR) at their respective cohort means and estimating change in the cognitive function Z-score at varying levels of albuminuria and eGFR. Given the exploratory nature of our analyses, the results were not adjusted for multiple testing, and *P* values <0.05 were considered nominally significant. All analyses were performed using SAS software (version 9.4).

## Results

Of the 1441 participants originally enrolled in DCCT, 1051 (83% of the surviving cohort) participated in cognitive testing at follow-up year 32 and were included in these analyses. Characteristics of the cohort at DCCT baseline, year 18, and year 32 are presented in Table 1. The mean age for this cohort at year 32 was 59 years, and 53% were male; 68% of participants had a history of hypertension, 76% had a history of hyperlipidemia, 26% had proliferative diabetic retinopathy, and 15% had a history of cardiovascular disease.

**Table 1.** Characteristics of Epidemiology of Diabetes Interventions and Complications participants with standardized cognition measurement at Diabetes Control and Complications Trial baseline and 18 and 32 years later^a^

Characteristic	DCCT Baseline	Study Year 18	Study Year 32
*N*	1051	940	1051
**Demographic**			
Intensive treatment assignment in DCCT	555 (53%)	500 (53%)	555 (53%)
Age, median (range)	27 (13–39)	46 (29–61)	59 (43–75)
Sex (male)	556 (53%)	510 (54%)	556 (53%)
White ethnicity (%)	1013 (96%)	909 (97%)	1013 (96%)
Education (yr)	14 (3)	16 (2)	16 (2)
Professional or technical occupation	333 (32%)	535 (57%)	577 (55%)
Verbal IQ	112 (11)	Not assessed	Not assessed
Full-scale IQ	114 (10)	Not assessed	Not assessed
**Nonglycemic characteristics**			
BMI, kg/m^2^	23.3 (2.8)	28.0 (4.7)	29.3 (5.8)
Systolic BP (mm Hg)	114 (12)	120 (13)	124 (15)
Diastolic BP (mm Hg)	72 (9)	74 (8)	69 (9)
History of hypertension^b^	33 (3%)	462 (50%)	715 (68%)
History of hyperlipidemia^c^	232 (22%)	445 (48%)	799 (76%)
**Glycemic characteristics/complications**			
HbA1c	8.7% (1.5)	7.7 (1.1)	7.8% (1.2)
Duration of diabetes, median (range), yr	4 (1–15)	24 (17–45)	37 (30–51)
Proliferative diabetic retinopathy	0	153 (16%)	275 (26%)
Cardiovascular disease^d^	0	42 (4%)	158 (15%)
**Medication usage**			
ACE inhibitors or ARB	0	390 (41%)	455 (43%)
Lipid-lowering medication	0	378 (40%)	742 (71%)
**Laboratory results**			
Total cholesterol (mg/dl)	175 (33)	179 (33)	171 (36)
HDL cholesterol (mg/dl)	51 (12)	54 (15)	64 (20)
LDL cholesterol (mg/dl)	109 (29)	107 (28)	92 (30)
Triglycerides (mg/dl)	78 (41)	86 (60)	74 (53)

DCCT, Diabetes Control and Complications Trial; IQ, intelligence quotient; BMI, body mass index; HbA1c, hemoglobin A1c; ACE, angiotensin-converting enzyme; ARB, angiotensin receptor blocker.

a Data are means (SD) or *n* (%), unless otherwise indicated.

b Defined as BP ≥140/90 mm Hg, documented hypertension, or use of antihypertensive medications.

c Defined as LDL cholesterol ≥130 mg/dl or use of lipid-lowering medications.

d As adjudicated by a committee masked to treatment group.

By design, there was very little kidney disease at DCCT baseline: 99% had an eGFR >90 ml/min per 1.73 m^2^, and 89.8% had normoalbuminuria (Table 2). However, by follow-up year 32, 69% of participants were classified as having persistent normoalbuminuria, while 8% had sustained AER 30 to <300 mg/24 hours, 12% had regressed AER 30 to <300 mg/24 hours, and 11% had AER >300 mg/24 hours. By follow-up year 32, eGFR also dropped, with only 43% of participants remaining above 90 ml/min per 1.73 m^2^, and 9% had an eGFR <60 ml/min per 1.73 m^2^.

**Table 2.** Distribution of eGFR and albuminuria categories over time in participants who had cognitive testing

Kidney Function	DCCT Baseline	Year 2	Year 5	Year 18	Year 32
*N*	1051	1040	826	940	1051
**eGFR at visit, ml/min per 1.73 m** ^**2**^					
<60	0	0	0	16 (2%)	96 (9%)
60 to <90	7 (1%)	11 (1%)	24 (3%)	187 (20%)	485 (46%)
≥90	1044 (99%)	1029 (99%)	802 (97%)	737 (78%)	470 (45%)
**Lowest attained eGFR, ml/min per 1.73 m** ^**2**^					
<60	0	0	0	12 (1%)	90 (9%)
60–74	4 (0.4%)	4 (0.4%)	3 (0.4%)	38 (4%)	173 (17%)
75–89	2 (0.2%)	4 (0.4%)	9 (1%)	143 (15%)	333 (32%)
≥90	1045 (99%)	1032 (99%)	814 (99%)	747 (80%)	455 (43%)
**AER, mg/24 h**					
<30	944 (90%)	921 (89%)	718 (87%)	811 (86%)	844 (80%)
30 to <300	107 (10%)	115 (11%)	96 (12%)	97 (10%)	158 (15%)
≥300	0	4 (0.4%)	12 (2%)	32 (3%)	49 (5%)
**Albuminuria status^a^**					
Persistent normoalbuminuria	1006 (96%)	954 (92%)	710 (86%)	722 (77%)	725 (69%)
Regressed AER 30-<300 mg/24 h	0	13 (1%)	31 (4%)	87 (9%)	123 (12%)
Sustained AER 30-<300 mg/24 h	45 (4%)	66 (6%)	69 (8%)	65 (7%)	86 (8%)
AER >300 mg/24 h	0	7 (1%)	16 (2%)	66 (7%)	117 (11%)

DCCT, Diabetes Control and Complications Trial; AER, albumin excretion rate.

a Normoalbuminuria: albumin excretion rate <30 mg/24 hours. Individuals were labeled as having persistent normoalbuminuria unless they developed sustained albumin excretion rate 30 to <300 mg/24 hours (persisted over two consecutive measurements) or albumin excretion rate >300 mg/24 hours. Those with sustained albumin excretion rate 30 to <300 mg/24 hours which then normalized on two consecutive measurements were considered to have regressed albumin excretion rate 30 to <300 mg/24 hours.

At follow-up year 32, mean (SD) Z-scores were −0.341 (0.899) for immediate memory, −0.278 (1.010) for delayed memory, and −1.311 (1.736) for psychomotor and mental efficiency. Changes in psychomotor efficiency Z-score over time by albuminuria and eGFR categories are shown in Figure 1. In fully adjusted longitudinal models, the strongest and most consistent associations were observed between markers of kidney function and psychomotor and mental efficiency and, to a lesser extent, immediate memory (Table 3). There were no significant associations between kidney function and delayed memory. For psychomotor and mental efficiency, albuminuria and, especially, eGFR were significantly associated with cognitive decline. Both were significant when modeled quantitatively, and more severe lowest attained eGFR showed a dose-response relationship with worsening Z-scores, as did lower eGFR at the study visit itself. For instance, lowest attained eGFR <60 ml/min per 1.73 m^2^ was associated with a 0.449 lower Z-score (95% confidence interval [CI], −0.640 to −0.259) for psychomotor and mental efficiency, equivalent to approximately 11 years of aging in this cohort on the basis of previous analyses.^8^ Sustained AER 30 to <300 mg/24 hours (*β* −0.148; 95% CI, −0.270 to −0.026), but not regressed AER 30 to <300 mg/24 hours (*β* 0.016; 95% CI, −0.126 to 0.158) or AER >300 mg/24 hours (*β* −0.018; 95% CI, −0.174 to 0.139), was correlated with lower psychomotor and mental efficiency domain Z-scores.

Figure 1.**Psychomotor efficiency Z-scores generally decline with worsened eGFR and albuminuria.** Change in psychomotor efficiency Z-score by (A) current eGFR, (B) lowest attained eGFR, (C) current AER, or (D) albuminuria category. AER, albumin excretion rate.

**Figure:**
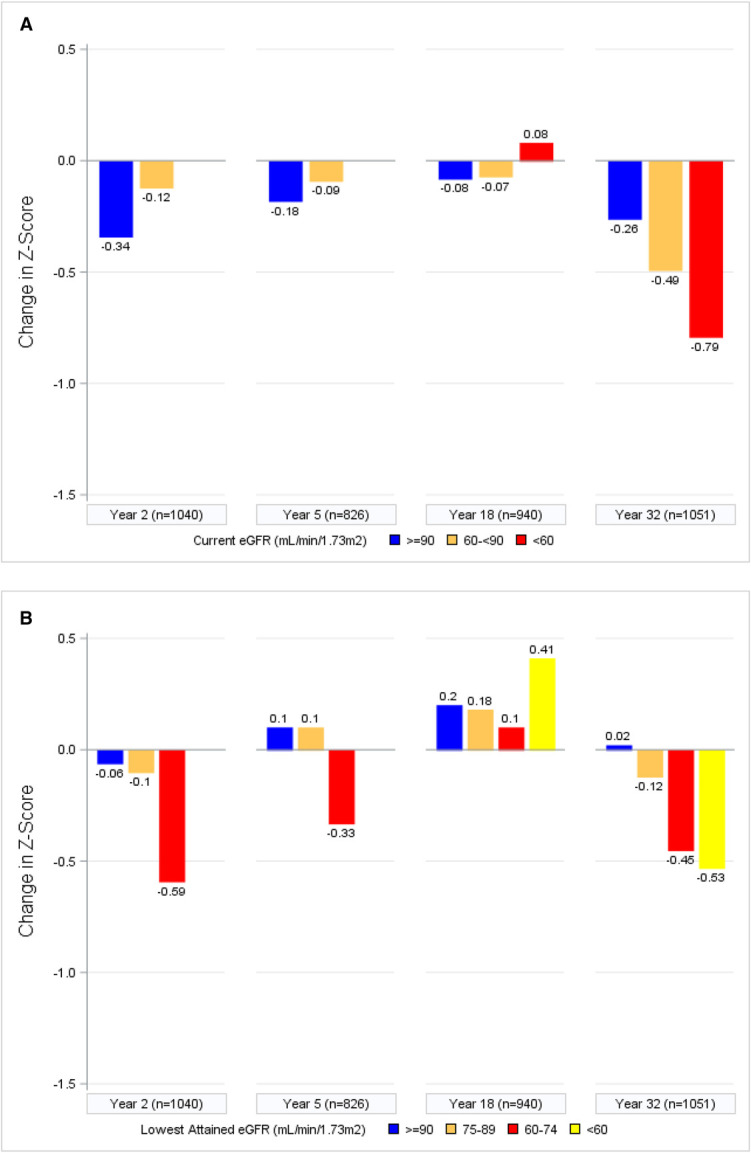

**Figure:**
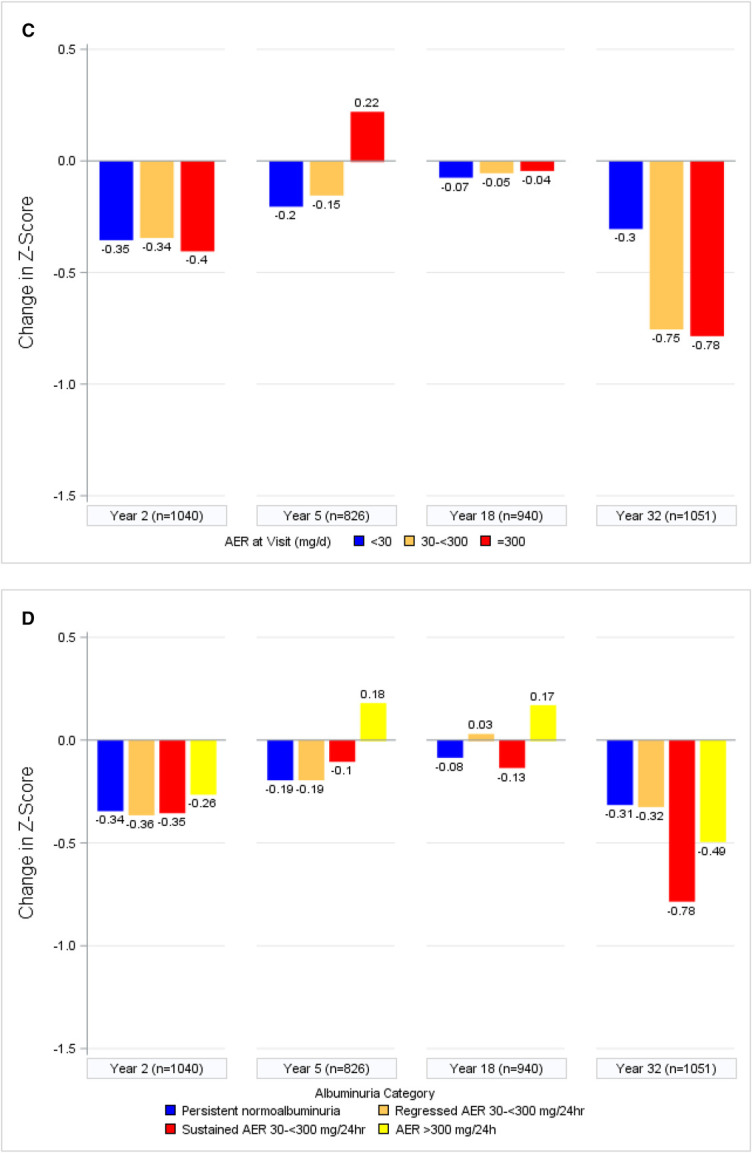

**Table 3.** Associations between albuminuria and eGFR with cognitive function in fully adjusted longitudinal models

Measurement	Immediate Memory^a^		Delayed Recall^b^		Psychomotor and Mental Efficiency^c^	
	*β* (95% CI)	*P* Value	*β* (95% CI)	*P* Value	*β* (95% CI)	*P* Value
Quantitative eGFR (per 15 ml/min per 1.73 m^2^ increase)	0.020 (−0.002 to 0.043)	0.077	0.027 (−0.001 to 0.056)	0.059	0.037 (0.003 to 0.070)^d^	0.035^d^
**eGFR at visit, ml/min per 1.73 m** ^**2**^		0.005^e^		0.062^e^		<0.001^e^
≥90	Ref	—	Ref	—	Ref	—
60 to <90	−0.031 (−0.089 to 0.026)	0.284	0.030 (−0.042 to 0.102)	0.408	−0.109 (−0.195 to −0.023)^d^	0.013^d^
<60	−0.199 (−0.318 to −0.080)	0.001^d^	**−**0.145 (**−**0.295 to 0.006)	0.060	−0.401 (−0.583 to −0.220)^d^	<0.001^d^
**Lowest attained eGFR, ml/min per 1.73 m** ^**2**^		0.058^e^		0.178^e^		<0.001^e^
≥90	Ref	—	Ref	—	Ref	—
75–89	**−**0.039 (**−**0.104 to 0.025)	0.233	0.005 (**−**0.076 to 0.085)	0.913	**−**0.070 (**−**0.166 to 0.025)	0.151
60–74	**−**0.095 (**−**0.187 to **−**0.002)	0.045^d^	**−**0.105 (**−**0.221 to 0.011)	0.077	−0.373 (−0.512 to −0.234)^d^	<0.001^d^
<60	**−**0.145 (**−**0.270 to **−**0.020)	0.023^d^	**−**0.107 (**−**0.265 to 0.050)	0.183	−0.449 (−0.640 to −0.259)^d^	<0.001^d^
Quantitative AER (per two-fold increase in AER)	0.004 (**−**0.008 to 0.017)	0.511	0.002 (**−**0.014 to 0.018)	0.827	−0.053 (−0.073 to −0.034)^d^	<0.001^d^
**AER at visit, mg/24 h**		0.021^e^		0.183^e^		0.009^e^
<30	Ref	—	Ref	—	Ref	—
30 to <300	**−**0.040 (**−**0.100 to 0.021)	0.200	**−**0.027 (**−**0.103 to 0.049)	0.491	−0.133 (−0.224 to −0.041)^d^	0.005^d^
≥300	0.149 (0.017 to 0.281)	0.028^d^	0.132 (**−**0.034 to 0.298)	0.120	**−**0.179 (**−**0.378 to 0.021)	0.080
**Albuminuria status^f^**		0.086^e^		0.458^e^		0.071^e^
Persistent normoalbuminuria	Ref	—	Ref	—	Ref	—
Regressed AER 30 to <300 mg/24 h	**−**0.036 (**−**0.135 to 0.063)	0.475	**−**0.050 (**−**0.176 to 0.076)	0.436	0.016 (**−**0.126 to 0.158)	0.827
Sustained AER 30 to <300 mg/24 h	**−**0.049 (**−**0.132 to 0.033)	0.243	**−**0.047 (**−**0.151 to 0.057)	0.378	−0.148 (−0.270 to −0.026)^d^	0.018^d^
AER >300 mg/24 h	0.096 (**−**0.008 to 0.199)	0.072	0.060 (**−**0.071 to 0.191)	0.373	**−**0.018 (**−**0.174 to 0.139)	0.824

*β* are reported as the estimated change in cognitive function Z-score compared with the overall cohort at Diabetes Control and Complications Trial baseline. Estimates are equal to the difference in means between categorical albuminuria or eGFR groups or per specified unit increase in continuous albumin excretion rate or eGFR. CI, confidence interval; AER, albumin excretion rate.

a Immediate memory domain: models adjusted for attained age, years of education.

b Delayed recall domain: models adjusted for attained age, years of education, and cardiovascular autonomic neuropathy.

c Psychomotor and mental efficiency domain: adjusted for attained age, sex, years of education, mean updated hemoglobin A1c, any hypoglycemia resulting in coma, mean updated systolic BP, mean updated pulse, any proliferative diabetic retinopathy, any clinically significant macular edema, any confirmed clinical neuropathy, any cardiovascular autonomic neuropathy, and any cardiovascular disease.

d statistically significant < 0.05.

e Associations between cognitive function and overall categorical albuminuria or eGFR groups were assessed using an F-test.

f Normoalbuminuria: albumin excretion rate <30 mg/24 hours. Individuals were labeled as having persistent normoalbuminuria unless they developed sustained albumin excretion rate 30 to <300 mg/24 hours (persisted over two consecutive measurements) or albumin excretion rate >300 mg/24 hours. Those with sustained albumin excretion rate 30 to <300 mg/24 hours which then normalized on two consecutive measurements were considered to have regressed albumin excretion rate 30 to <300 mg/24 hours.

Mean (SD) change in cognitive function from year 18 to year 32 was −0.297 (0.723) for immediate memory, −0.440 (0.793) for delayed memory, and −1.071 (1.283) for psychomotor and mental efficiency. In fully adjusted models, we found that kidney function at year 18 was associated with a significant decline in psychomotor and mental efficiency between study years 18 and 32 (Table 4). Specifically, the lowest attained eGFR <60 ml/min per 1.73 m^2^ (*β* −0.915; 95% CI, −1.613 to −0.217), eGFR <60 ml/min per 1.73 m^2^ at year 18 (*β* −0.886; 95% CI, −1.493 to −0.279), and AER 30 to <300 mg/24 hours at year 18 (*β* −0.271; 95% CI, −0.538 to −0.004) were each independently associated with lower psychomotor and mental efficiency domain Z-scores at study year 32. Sustained AER 30 to <300 mg/24 hours and AER >300 mg/24 hours were not significantly associated with changes in psychomotor and mental efficiency, and no marker of kidney function was associated with changes in immediate or delayed memory between years 18 and 32. Similar analyses were conducted evaluating kidney function at year 5 in relation to changes in cognition between follow-up years 5 and 18, yielding no significant associations (Supplemental Table 2).

**Table 4.** Associations between kidney function at year 18 and cognitive change between year 18 and year 32

Measurement	Immediate Memory^a^		Delayed Recall^b^		Psychomotor and Mental Efficiency^c^	
	*β* (95% CI)	*P* Value	*β* (95% CI)	*P* Value	*β* (95% CI)	*P* Value
Quantitative eGFR (per 15 ml/min per 1.73 m^2^)	**−**0.002 (**−**0.051 to 0.048)	0.949	0.016 (**−**0.038 to 0.070)	0.567	0.069 (**−**0.017 to 0.154)	0.116
**eGFR at visit, ml/min per 1.73 m** ^**2**^		0.917^d^		0.358^d^		0.016^d^
≥90	Ref	—	Ref	—	Ref	—
60 to <90	**−**0.016 (**−**0.137 to 0.105)	0.795	**−**0.096 (**−**0.228 to 0.037)	0.157	**−**0.061 (**−**0.266 to 0.145)	0.561
<60	0.055 (**−**0.302 to 0.413)	0.762	0.023 (**−**0.370 to 0.415)	0.910	−0.886 (−1.493 to −0.279)^e^	0.004^e^
**Lowest attained eGFR, ml/min per 1.73 m** ^**2**^		0.662^d^		0.732^d^		0.024^d^
≥90	Ref	—	Ref	—	Ref	—
75–89	**−**0.038 (**−**0.172 to 0.096)	0.583	**−**0.066 (**−**0.214 to 0.082)	0.381	**−**0.124 (**−**0.349 to 0.102)	0.284
60–74	0.046 (**−**0.192 to 0.285)	0.703	0.024 (**−**0.240 to 0.288)	0.857	0.236 (**−**0.174 to 0.647)	0.260
<60	0.213 (**−**0.198 to 0.624)	0.310	0.142 (**−**0.310 to 0.594)	0.538	−0.915 (−1.613 to −0.217)^e^	0.010^e^
Quantitative AER (per two-fold increase in AER)	**−**0.005 (**−**0.031 to 0.022)	0.730	**−**0.006 (**−**0.035 to 0.023)	0.691	**−**0.033 (**−**0.084 to 0.017)	0.198
**AER at visit, mg/d**		0.908^d^		0.746^d^		0.139^d^
<30	Ref	—	Ref	—	Ref	—
30 to <300	**−**0.033 (**−**0.185 to 0.120)	0.675	0.036 (**−**0.131 to 0.203)	0.675	−0.271 (−0.538 to −0.004)^e^	0.047^e^
≥300	0.014 (**−**0.241 to 0.268)	0.917	**−**0.087 (**−**0.367 to 0.193)	0.543	**−**0.051 (**−**0.517 to 0.414)	0.829
**Albuminuria status^f^**		0.836^d^		0.258^d^		0.795^d^
Persistent normoalbuminuria	Ref	—	Ref	—	Ref	—
Regressed AER 30 to <300 mg/24 h	**−**0.006 (**−**0.167 to 0.156)	0.945	0.095 (**−**0.084 to 0.274)	0.299	**−**0.107 (**−**0.388 to 0.174)	0.457
Sustained AER 30 to <300 mg/24 h	0.083 (**−**0.100 to 0.267)	0.374	0.166 (**−**0.035 to 0.367)	0.107	**−**0.129 (**−**0.451 to 0.194)	0.435
AER >300 mg/24 h	**−**0.012 (**−**0.195 to 0.170)	0.894	**−**0.057 (**−**0.259 to 0.144)	0.577	**−**0.044 (**−**0.388 to 0.301)	0.805

*β* are reported as the estimated change in cognitive function Z-score between study years 18 and 32 compared with the overall cohort at Diabetes Control and Complications Trial baseline. Estimates are equal to the difference in means between categorical albuminuria or eGFR groups or per specified unit increase in continuous albumin excretion rate or eGFR. CI, confidence interval; AER, albumin excretion rate.

a Immediate memory domain: models adjusted for attained age, years of education.

b Delayed recall domain: models adjusted for attained age, years of education, and cardiovascular autonomic neuropathy.

c Psychomotor and mental efficiency domain: adjusted for attained age, sex, years of education, mean updated hemoglobin A1c, any hypoglycemia resulting in coma, mean updated systolic BP, mean updated pulse, any proliferative diabetic retinopathy, any clinically significant macular edema, any confirmed clinical neuropathy, any cardiovascular autonomic neuropathy, and any cardiovascular disease.

d Associations between cognitive function and overall categorical albuminuria or eGFR groups were assessed using an F-test.

e statistically significant < 0.05.

f Normoalbuminuria: albumin excretion rate <30 mg/24 hours. Those with sustained albumin excretion rate 30 to <300 mg/24 hours which then normalized on two consecutive measurements were considered to have regressed albumin excretion rate 30 to <300 mg/24 hours.

**Figure 2.**
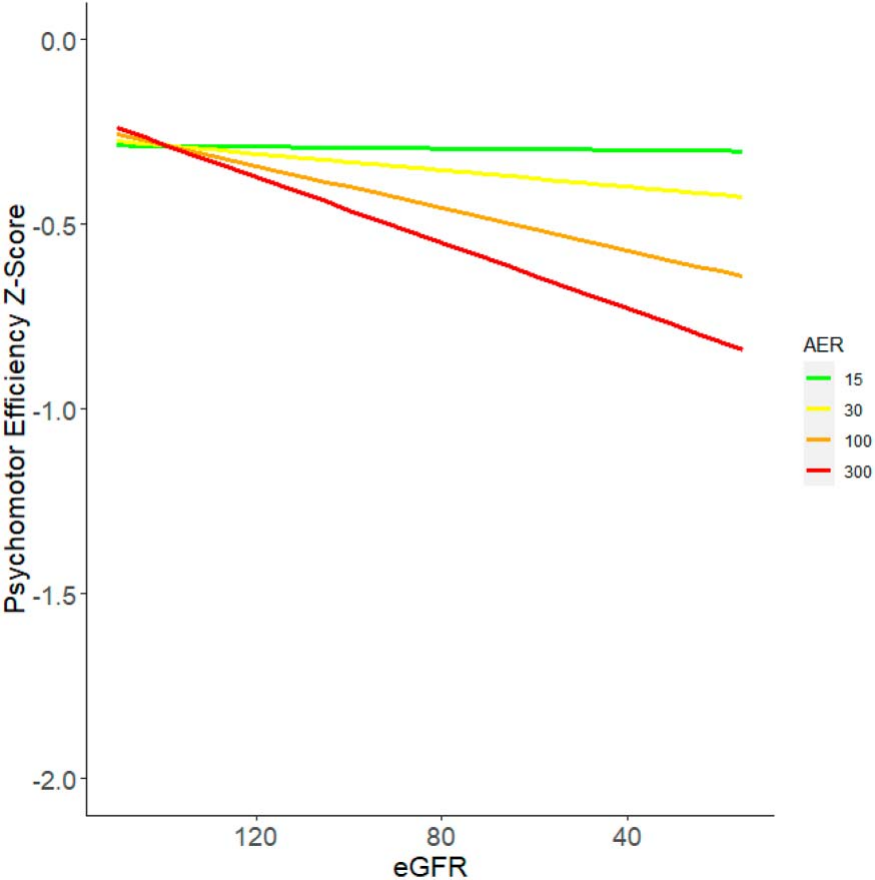
**Combination of worsened albuminuria and eGFR associate with worsened psychomotor efficiency z-score.** Estimated change in psychomotor and mental efficiency Z-scores by eGFR at various levels of AER—fully adjusted using data from all measurements. Estimated change in Z-score obtained from a longitudinal mixed model adjusting for age, sex, years of education, mean updated HbA1c, any hypoglycemia resulting in coma, mean updated systolic BP, mean updated pulse, any proliferative diabetic retinopathy, any clinically significant macular edema, any confirmed clinical neuropathy, any cardiovascular autonomic neuropathy, any cardiovascular disease, and interaction between AER and eGFR. Estimates are calculated by fixing all covariates except AER and eGFR at their respective cohort means, AER at 15, 30, 100, 300 mg/24 hours, and varying eGFR. *P for interaction=0.003. AER, albumin excretion rate; HbA1c, hemoglobin A1c.

Figure 2 illustrates the interconnected relationship between AER and eGFR for longitudinal changes in the psychomotor and mental efficiency domain. Participants with the lowest eGFR and highest AER had the most significant declines in cognitive function while participants with normoalbuminuria did not show any worsening in cognitive function (*P* for interaction 0.003).

## Discussion

In this longitudinal study of participants with T1D, the presence of kidney disease was significantly associated with cognitive decline over 32 years of follow-up, particularly within the psychomotor and mental efficiency domain. Associations were independent of known potential confounders such as age, glycemic control, and hypertension. These results suggest that the development of kidney disease, a common complication of T1D, may accelerate subsequent cognitive decline in this population.

Our findings augment prior reports in the field of CKD and cognition. Among those with diabetes, the vast majority of literature pertaining to CKD and cognitive decline has focused on type 2 diabetes^29^ with limited data regarding T1D.^30^ To the best of our knowledge, this is the most comprehensive epidemiological assessment of the kidney–brain axis in people with T1D. Moreover, whereas most analyses have investigated populations where CKD was already established, our results document cognition function both before and after the onset of kidney disease, thus strengthening the independent temporal association observed. We evaluated the relationship between CKD and changing cognitive scores over a lengthy period of time in a longitudinal assessment, which has also been lacking in many previous studies with shorter follow-up. In our analyses, kidney function up to follow-up year 5 was not associated with cognitive changes at year 18. On the other hand, both eGFR <60 ml/min per 1.73 m^2^ and sustained AER 30 to <300 mg/24 hours up to year 18 were associated with significant declines in psychomotor and mental efficiency at year 32. The difference in these findings may be due to the low prevalence and relatively short duration of CKD as well as a less pronounced change in overall cognitive function during the earlier years of the study.

Prior evidence strongly suggests that more advanced CKD, particularly eGFR <45 ml/min per 1.73 m^2^ or ESKD, is associated with reduced cognitive function, regardless of CKD etiology.^31–33^ However most, though not all, studies have shown similar associations with milder kidney disease.^6,34–38^ In this study, we were able to capture changes in CKD status over time both in eGFR and albuminuria, a unique advantage over other cohorts studied, and we demonstrated that adverse cognitive effects may begin with eGFR as high as 60–74 ml/min per 1.73 m^2^. We found a dose-response relationship between worsened categories of eGFR and cognitive function consistent with other studies in the general population.^5^ We also demonstrated associations with AER 30 to <300 mg/24 hours and cognitive decline, although an adverse signal with AER >300 mg/24 hours was not observed. This may be due to the relatively small number of people who developed AER >300 mg/24 hours because we still showed a negative relationship with quantitatively modeled albuminuria, but this requires further study. In contrast to the negative associations with sustained AER 30 to <300 mg/24 hours, we did not find a correlation with regressed AER 30 to <300 mg/24 hours. This raises the possibility that more aggressive treatment of AER 30 to <300 mg/24 hours may have beneficial cognitive effects. This was also suggested by secondary analyses of the Ongoing Telmisartan Alone and in Combination With Ramipril Global End Point Trial and Telmisartan Randomized Assessment Study in Angiotensin-Converting Enzyme–Intolerant Subjects With Cardiovascular Disease studies, where treatment with either angiotensin receptor blocker or an angiotensin-converting enzyme inhibitor among those with severely elevated albuminuria attenuated odds of cognitive decline.^39^

Our results pertaining to albuminuria are in line with several other studies,^40–43^ although divergent from results of the Systolic Blood Pressure Intervention Trial, which did not include patients with diabetes, where baseline and incident albuminuria were not associated with dementia or mild cognitive impairment.^44^ The Reasons for Geographic and Racial Disparities in Stroke (REGARDS) study also did not find an association between albuminuria and cognitive decline at lower eGFR.^45^ On the contrary, an analysis of the Atherosclerosis Risk in Communities cohort found that the highest risk of dementia was in those with lower eGFR and higher albuminuria.^46^ Differences in study design, duration of follow-up, and population may account for these discordant findings. The REGARDS trial had a shorter follow-up period with fewer measurements of albuminuria, an older demographic, and a significant proportion of participants without diabetes. It is possible that in the older REGARDS population, eGFR may have been overestimated in some participants, reducing the effect of albuminuria at lower eGFR. The diabetic phenotype may also carry additional risks at the microvascular level, reflected by high degrees of proteinuria, which may not be the case with other forms of kidney disease. We measured albuminuria at many time points over 30+ years, enabling a more complete assessment of each individual's albuminuria burden. This comprehensive evaluation demonstrated that higher levels of albuminuria at any given eGFR were associated with greater cognitive impairment, consistent with risks of cardiovascular disease and mortality in the CKD population.^47^ Defining the exact nature of the interaction between albuminuria and reduced eGFR for cognitive decline clearly is an area that needs further study.

It is impossible to determine causality from observational data; however, there are numerous unique plausible mechanisms, whereby kidney function may cause deterioration in cognitive function. Individuals with CKD have been shown to carry an increased burden of structural cerebral changes including white matter disease,^3^ microbleeds,^48^ and atrophy.^2^ These findings may correlate with impaired microvascular health and endothelial dysfunction, which are closely tied to CKD and albuminuria.^5,49^ Certain uremic toxins, such as indoxyl sulfate, seen in more advanced CKD are more likely to cross the blood–brain barrier and have been negatively associated with cognitive function in hemodialysis patients.^50^ One of the hallmarks of CKD is a deranged mineral metabolism axis. Reduced levels of circulating *α*-klotho^51^ and elevated levels of fibroblast growth factor 23^52^ and parathyroid hormone^53^ have all been linked to cognitive impairment and can be seen with even early CKD.^54,55^ In addition, the proinflammatory state accompanying CKD may negatively affect cognition through various mechanisms, including modulating neural progenitor cells.^5^ Finally, those with CKD have a clustering of other risk factors that may lead to cognitive changes. Nevertheless, we adjusted for numerous confounders and still found strong independent association between kidney function and cognition despite the relatively lower burden of comorbidities such as cardiovascular disease and obesity, compared with other cohorts that reported relationships between CKD and cognitive function.

In our study, the associations between CKD and cognition were most pronounced with psychomotor and mental efficiency, the domain most closely linked to the detrimental effects of hyperglycemia.^8^ Although these declines may be modest, poorer performance on tests like these has been associated with difficulty performing tasks such as meal preparation^56^ and driving.^57^ Previous studies in CKD that focused on specific cognitive domains have been limited with small sample sizes and cross-sectional data. In general, executive dysfunction including impaired Trail Making Test scores^58,59^ is found to a more significant extent in those with CKD, as opposed to less affected domains such as language and visuospatial performance.^5,60^ This is consistent with imaging findings of relative frontal lobe atrophy among those with CKD.^61^ Memory may also be affected by CKD, although we found no association between kidney function and delayed memory. By contrast, lowest attained eGFR <74 ml/min per 1.73 m^2^ was associated with poorer immediate memory. It is possible that memory may be relatively preserved in mild CKD and may decline as a consequence of more advanced kidney disease, as has been suggested by other reports.^62^

There are numerous strengths to this analysis. First, the DCCT/EDIC study offers longitudinal data over 30 years with limited loss to follow-up in a large, well-characterized cohort of participants with long-standing T1D diabetes. Second, a battery of validated cognitive testing was performed repeatedly over time, creating a robust cognitive profile for each participant. Third, data were collected repeatedly throughout the study period with consistent, standardized measurements, thereby allowing us to account for changing kidney function and other covariates, with adjustment for an extensive array of potential confounders. We were also able to study cognitive function uniquely because it pertains to CKD both before and after the onset of kidney disease, given the essentially normal kidney function of most participants at baseline. However, there are also limitations to this study. The overall severity of kidney disease in this cohort was modest, thereby limiting generalizability to more advanced CKD. Nevertheless, we did find a dose-dependent relationship with less severe derangements in kidney function, suggesting that more severe CKD could correlate with more cognitive dysfunction in this population, as has been illustrated in other studies not focused on T1D. Finally, this cohort is mostly White and with relatively higher baseline level of education, and these findings should be replicated in other populations.

We found that mild-to-moderate CKD in T1D is associated with long-term cognitive decline, independent of glycemic control and other potential confounding factors. This highlights the importance of CKD screening, prevention, and treatment to potentially reduce risks of cognitive decline in this vulnerable population. Individualized time frames for albuminuria testing have been proposed in type 1 diabetes mellitus,^63^ and its potential links to cognitive function may provide further motivation for precision screening, especially considering the favorable effects of regressed albuminuria (versus persistent albuminuria) seen in this cohort. Increased education of patients, families, and clinicians to better understand implications of CKD and ability to influence renal-related outcomes would also be beneficial. Further research is needed to determine pathogenesis, unravel any interactions between albuminuria and eGFR at lower levels of eGFR, and potential benefits of early treatment of albuminuria to slow cognitive decline.

## Supplementary Material

SUPPLEMENTARY MATERIAL

## Data Availability

Data collected for the DCCT/EDIC study through June 30, 2017, are available to the public through the NIDDK Central Repository (https://repository.niddk.nih.gov/studies/edic/). Data collected in the current cycle (July 2017–June 2022) will be available within 2 years after the end of the funding cycle.

## Trial Registration

ClinicalTrials.gov: NCT00360815 and NCT00360893.

## Supplemental Material

Supplemental Table 1. Description of tests by cognitive domain.

Supplemental Table 2. Effect of year 5 measurements on cognitive function change between year 5 and year 18 in fully adjusted models.
