# Supplementary material for: CKD Associates with Cognitive Decline in Middle-Aged and Older Adults with Long-Standing Type 1 Diabetes
Source: Kidney360. 2023 Jun 9;4(8):1058–71. doi: 10.34067/KID.0000000000000178 (PMC10476689; doi:10.34067/KID.0000000000000178)
Supplement: SUPPLEMENTARY MATERIAL [file kidney360-4-1058-s001.pdf]

Supplemental Table 1: Description of Tests by Cognitive Domain

**Immediate Memory**		
Logical Memory Subtest from Wechsler Memory Scale	Recall each of 2 stories immediately after hearing them	Total number of 'idea units' recalled (maximum = 45)
Digit Symbol Recall	Recall of the 9 digit/symbol pairs used in Digit-Symbol Substitution Test (DSST)	Total correct (maximum = 9)
**Delayed Memory**		
Logical Memory Subtest from Wechsler Memory Scale	Recall each of 2 stories after a 10-15 minute delay	Total number of 'idea units' recalled (maximum = 45)
**Psychomotor and Mental Efficiency**		
Verbal Fluency	Generate 3 lists of words, beginning with 'F,' 'A,' and 'S' respectively in 3 60-second trials	Total number of words generated
Digit Symbol Substitution Test from Wechsler Adult Intelligence Scale	Use a digit-symbol code to replace 9 numbers for letters as quickly as possible	Total time to complete the entire 90 item grid (higher score reflects poorer performance)
Trail Making (Part B)	Scan page of numbers (1-13) and letters (A-L) and draw line that alternates between them in consecutive order (1-A-2-B…)	Total time to complete task (higher score reflects poorer performance)
Grooved Pegboard	Key-shaped pegs are inserted in board with 25 keyholes as quickly as possible, first with dominant, then non-dominant, hand	Total time to complete task (higher score reflects poorer performance)

Supplemental Table 2: Effect of Year 5 Measurements on Cognitive Function Change Between Year 5 and Year 18 in Fully Adjusted Models

	Immediate Memory*		Delayed Recall**		Psychomotor and mental efficiency***	
Measurement	Beta (95% CI)	P-Value	Beta (95% CI)	P-Value	Beta (95% CI)	P-Value
**Quantitative eGFR (per 15 ml/min/1.73m²)**	0.021 (-0.043, 0.086)	0.515	-0.024 (-0.109, 0.062)	0.589	-0.035 (-0.094, 0.024)	0.242
**eGFR at Visit**		0.358^		0.589^		0.142^
≥90 mL/min/1.73m²	ref	-	ref	-	ref	-
60-<90 mL/min/1.73m²	-0.128 (-0.402, 0.145)	0.358	-0.115 (-0.477, 0.247)	0.535	0.186 (-0.062, 0.433)	0.142
<60 mL/min/1.73m² (#)	n/a		n/a		n/a	
**Lowest Attained eGFR**		0.461^		0.721^		**0.040^**
≥90 mL/min/1.73m²	ref	-	ref	-	ref	-
75-89 mL/min/1.73m²	-0.287 (-0.739, 0.166)	0.215	0.145 (-0.454, 0.745)	0.635	**0.476 (0.068, 0.885)**	**0.023**
60-74 mL/min/1.73m²	-0.051 (-0.780, 0.678)	0.890	-0.317 (-1.283, 0.648)	0.519	0.383 (-0.273, 1.039)	0.253
<60 mL/min/1.73m² (#)	n/a		n/a		n/a	
**Quantitative AER (per two-fold increase in AER)**	0.003 (-0.030, 0.036)	0.850	-0.008 (-0.052, 0.036)	0.727	0.015 (-0.018, 0.047)	0.375
**AER at Visit**		0.773^		0.061^		0.758^
<30 mg/24hr	ref	-	ref	-	ref	-
30-<300 mg/24hr	0.033 (-0.112, 0.178)	0.658	-0.173 (-0.366, 0.019)	0.077	0.030 (-0.107, 0.167)	0.668
≥300 mg/24hr	0.115 (-0.268, 0.497)	0.558	0.378 (-0.129, 0.886)	0.144	0.123 (-0.246, 0.491)	0.514
**Albuminuria Status**		0.353^		0.114^		0.675^
Persistent normoalbuminuria	ref	-	ref	-	ref	-
Regressed AER 30-<300 mg/24hr	0.176 (-0.058, 0.411)	0.141	-0.079 (-0.395, 0.237)	0.625	0.020 (-0.197, 0.237)	0.859
Sustained AER 30-<300 mg/24hr	0.065 (-0.103, 0.234)	0.447	-0.217 (-0.441, 0.007)	0.058	0.063 (-0.096, 0.221)	0.438
AER >300 mg/24h	0.150 (-0.179, 0.478)	0.372	0.302 (-0.135, 0.739)	0.176	0.168 (-0.147, 0.483)	0.297

Betas are reported as the estimated change in cognitive function z-score between study years 5 and 18 compared to the overall cohort at DCCT baseline. Estimates are equal to the difference in means between categorical albuminuria or eGFR groups, or per specified unit increase in continuous AER or eGFR.

^ Associations between cognitive function and overall categorical albuminuria or eGFR groups were assessed using an F-test.

* Immediate memory domain: models adjusted for attained age, years of education

** Delayed recall domain: models adjusted for attained age, years of education, and cardiovascular autonomic neuropathy.

*** Psychomotor and mental efficiency domain: adjusted for attained age, sex, years of education, mean updated HbA1c, any hypoglycemia resulting in coma, mean updated systolic blood pressure, mean updated pulse, any PDR, any CSME, any confirmed clinical neuropathy, any cardiovascular autonomic neuropathy, and any cardiovascular disease.

There were no participants at DCCT year 5 with eGFR < 60 mL/min/1.73m²

Abbreviations: AER (albumin excretion rate); eGFR (estimated glomerular filtration rate)
